# Guidelines for *de novo* phasing using multiple small-wedge data collection

**DOI:** 10.1107/S1600577521008067

**Published:** 2021-08-26

**Authors:** Seiki Baba, Hiroaki Matsuura, Takashi Kawamura, Naoki Sakai, Yuki Nakamura, Yoshiaki Kawano, Nobuhiro Mizuno, Takashi Kumasaka, Masaki Yamamoto, Kunio Hirata

**Affiliations:** a Protein Crystal Analysis Division, Japan Synchrotron Radiation Research Institute, 1-1-1 Kouto, Sayo, Hyogo 679-5198, Japan; b Life Science Research Infrastructure Group, RIKEN SPring-8 Center, 1-1-1 Kouto, Sayo-Cho, Sayo-gun, Hyogo 679-5148, Japan

**Keywords:** small-wedge synchrotron crystallography (SWSX), protein crystallography, radiation damage, *de novo* phasing, dose

## Abstract

Intense micro-focus X-ray beamlines available at synchrotron facilities have achieved high-quality data collection even from the microcrystals of membrane proteins. The automatic data collection system developed at SPring-8, named ZOO, has contributed to many structure determinations of membrane proteins using small-wedge synchrotron crystallography (SWSX) datasets. The ‘small-wedge’ (5–20°) datasets are collected from multiple crystals and then merged to obtain the final structure factors. To our knowledge, no systematic investigation on the dose dependence of data accuracy has so far been reported for SWSX, which is between ‘serial crystallography’ and ‘rotation crystallography’. Thus, herein, we investigated the optimal dose conditions for experimental phasing with SWSX. Phase determination using anomalous scattering signals was found to be more difficult at higher doses. Furthermore, merging more homogeneous datasets grouped by hierarchical clustering with controlled doses mildly reduced the negative factors in data collection, such as ‘lack of signal’ and ‘radiation damage’. In turn, as more datasets were merged, more probable phases could be obtained across a wider range of doses. Therefore, our findings show that it is essential to choose a lower dose than 10 MGy for *de novo* structure determination by SWSX. In particular, data collection using a dose of 5 MGy proved to be optimal in balancing the amount of signal available while reducing the amount of damage as much as possible.

## Abstract 2

A guideline for *de novo* phasing with small-wedge synchrotron crystallography data collection is presented based on systematic investigations of dose and number of merged sub-datasets.

## 1. Introduction   

Recently, it has become possible to accomplish structural analysis even from small crystals with weak diffracting power, such as membrane proteins. High-brilliance and small X-ray beams available at synchrotron radiation facilities enable reduced scattering from the noncrystalline volume and measurement of weak diffraction intensities from tiny crystals with high signal-to-noise ratio (Smith *et al.*, 2012 ▸; Owen *et al.*, 2017 ▸; Yamamoto *et al.*, 2017 ▸). In addition, the combination of two-dimensional detectors with fast readout time, faster computers and more sophisticated analysis methods has dramatically improved the efficiency of data measurement and analysis (Holton & Alber, 2004 ▸; Panjikar *et al.*, 2005 ▸; Kabsch, 2010 ▸; van den Bedem *et al.*, 2011 ▸; Monaco *et al.*, 2013 ▸; Winter *et al.*, 2018 ▸; Basu *et al.*, 2019 ▸). Nonetheless, radiation damage is still a limitation for data collection from tiny protein crystals since the amount of signal per absorbed dose is reduced as the crystal volume diminishes (Holton & Frankel, 2010 ▸). In particular, *in meso* crystals of membrane proteins are significantly more difficult to grow at increased size, and the intrinsic diffraction ability of individual crystals tends to be lower. Furthermore, when crystals are harvested from the glass sandwich plate, they are sometimes retrieved in 20–30 s to reduce degradation of crystals due to phase transition of lipids or drying of the media. Hence, researchers need to quickly mount multiple crystals on a loop with poor control on how the crystals are mounted, in contrast to conventional rotation crystallography. Small-wedge synchrotron crystallography (SWSX) was developed to solve these problems (Cherezov *et al.*, 2007 ▸; Rasmussen, Choi *et al.*, 2011 ▸; Rosenbaum *et al.*, 2011 ▸).

**Figure 1:**
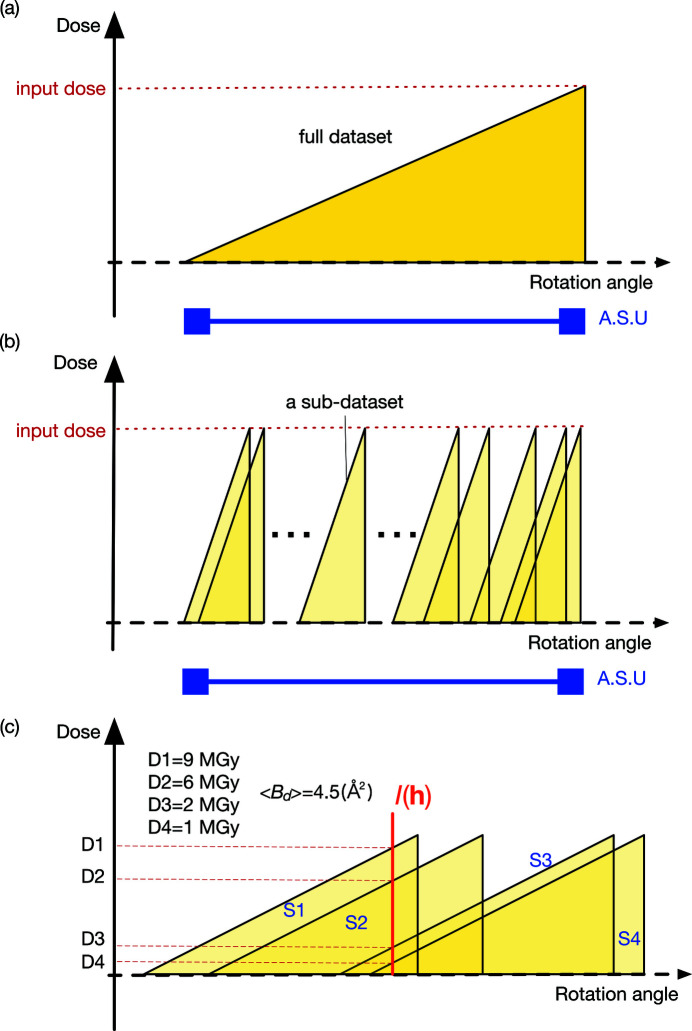
Schematic diagrams of dose-controlled data collection protocols implemented in the ZOO system: (*a*) normal rotation method, (*b*) small wedge synchrotron crystallography (SWSX). The ‘input dose’ is a user-defined dose for data collection. (*c*) How to estimate 〈*B*_d_〉 for a particular reflection intensity, *I*(**h**), from redundant measurements using multiple crystals in SWSX. A.S.U. in (*a*) and (*b*) indicates an asymmetric unit of the reciprocal space. Note that the scale of rotation angle is different in (*c*) than in (*a*) and (*b*). D1–D4 in (*c*) indicates the dose values at which the intensity was observed for sub-datasets S1–S4.

In SWSX, multiple small-wedge (5–20°/crystal) datasets (hereafter referred to as sub-datasets) are collected from tens or hundreds of crystals in different orientations, which are then merged to obtain a complete dataset. A cryo-loop with multiple crystals is raster-scanned with X-rays (Cherezov *et al.*, 2009 ▸; Aishima *et al.*, 2010 ▸; Zander *et al.*, 2015 ▸), and sub-datasets are collected from each crystal, assuming that the crystal is located at the position in which diffraction is observed in the raster scan. In conventional rotation data collection, each crystal is aligned so that it remains in irradiation by the X-ray beam regardless of its rotation. In contrast, in SWSX, the wedge size must be small to avoid misalignment between the beam and the crystal, so the wedge size is commonly set within 5–20° for each crystal; however, this specification depends on the beam and crystal sizes. Measuring multiple sub-datasets from a single loop facilitates data acquisition from a large number of crystals. Furthermore, SWSX is compatible with the currently available microbeam measurement schemes and dramatically improves the efficiency of the crystal harvest, since it does not require the microcrystals to be captured one by one by the cryo-loop. Finally, a large number of sub-datasets are merged to produce a highly complete dataset after individual data reduction. In SWSX, the same dose is administered deliberately to a single small wedge of a sub-dataset as is absorbed during a full rotation data set [Figs. 1 ▸(*a*) and 1(*b*)], so that SWSX provides highly multiplicitous data with a higher signal-to-noise ratio than traditional rotation crystallography.

Various developments have been made in data processing and analysis methods for merging data collected from multiple crystals for structural analysis. Hierarchical clustering of sub-datasets based on correlation of unit-cell parameters or intensities, and selection of merged data with several algorithms have been proposed (Giordano *et al.*, 2012 ▸; Foadi *et al.*, 2013 ▸; Zander *et al.*, 2016 ▸; Assmann *et al.*, 2020 ▸; Kovalenko *et al.*, 2020 ▸). These methods have enabled more efficient and accurate selection and merging of good datasets from a large number of sub-datasets. Performing such serial operations in SWSX manually is prone to human error and delay. Therefore, efforts have been made to eliminate manual operations as much as possible and to automate each process to promote efficient structure analysis at the macromolecular crystallography (MX) beamlines at synchrotron facilities. Nowadays, facilities for automated measurements at synchrotrons are becoming increasingly available (Bowler *et al.*, 2015 ▸; Svensson *et al.*, 2015 ▸; Zander *et al.*, 2015 ▸).

At the MX beamlines of SPring-8, we have developed an automated data collection system using a high-brightness microbeam, named ZOO, to fully automate goniometer-based data collection, including SWSX (Hirata *et al.*, 2019 ▸). The beamline instrumentation consists of a computer-controlled diffractometer, a high-speed sample changer (Murakami *et al.*, 2020 ▸), and a large-area pixel array detector. A device to automatically remove ice from crystals is also implemented and available before the data collection (Nakamura *et al.*, 2020 ▸). The SPring-8 MX beamline control software (BSS) provides the precise control required for diffraction experiments, adjustment of optics, change of beam size, and alignment of the X-ray beam position (Ueno *et al.*, 2005 ▸). The ZOO system realizes fully automated data acquisition along with this equipment and the control software. It implements full automation of all possible goniometer-based data collection, such as rotation data collection, helical data collection, SWSX, and serial synchrotron rotation crystallography experiments (Hasegawa *et al.*, 2017 ▸). The system also realizes dose-controlled data collection as determined by the users. Based on our experience, a dose of 10 MGy is recommended for native data collection, whereas for phase determination 5 MGy is recommended for any implemented schemes in ZOO. After the data measurement, *KAMO* automatically proceeds with the data processing for each wedge, performing hierarchical clustering, and automatic merging for each clustering node (Yamashita *et al.*, 2018 ▸). Hence, ZOO is effective for measuring large numbers of microcrystals.

The results achieved by ZOO have successfully proven the effectiveness of SWSX in the structural analysis of various samples at SPring-8 (Taniguchi *et al.*, 2017 ▸; Kato *et al.*, 2018 ▸; Shihoya *et al.*, 2018 ▸; Liu *et al.*, 2019 ▸; Ikuta *et al.*, 2020 ▸; Jiang *et al.*, 2020 ▸; Shiimura *et al.*, 2020 ▸; Umeda *et al.*, 2020 ▸; Yu *et al.*, 2020 ▸). In these studies, hierarchical clustering was shown to be essential to select homogeneous sub-datasets to be merged. However, there has been no systematic study assessing SWSX data analysis as a function of the absorbed dose.

Herein, we conducted experiments to evaluate the optimal dose for SWSX using microcrystals in order to efficiently collect highly accurate data. Lysozyme crystals of controlled size were used as evaluation samples and the optimal dose to obtain highly accurate data was investigated by sulfur-single-wavelength anomalous diffraction (S-SAD) phasing. To mimic the *in meso* experiments, we conducted SWSX data collection using 20 µm-sized lysozyme crystals on a cryo-loop in a dense configuration. Here, the SWSX was different from the measurement method that aligns the crystal to the center of rotation of the goniometer. The wedge size for data collection was set to 10°/crystal, which is the default for SWSX in ZOO measurement based on our extensive experience. Supported by experimental results, we discuss the effect of merging a larger number of sub-datasets and the appropriate dose setting for *de novo* phasing. By merging more sub-datasets, the effects of signal summation, random error reduction, and even ‘apparent dose reduction’ were observed. In addition, we will introduce the practical phase determination of membrane proteins with SWSX facilitated by hierarchical clustering. The collected data and our simulations emphasize that it is extremely important to control the maximum dose used for SWSX to efficiently enhance the selection and analysis accuracy of the datasets.

## 2. Materials and methods   

### 2.1. Preparation of lysozyme microcrystals   

Lysozyme (#L6876-5G; Lot: SLBT5180; Sigma-Aldrich, St Louis, MO, USA) was dissolved in 10 m*M* acetic acid (pH 4.6) to prepare a 40 mg ml^−1^ solution. Lysozyme and precipitant {4 *M* Na-malonate (pH 3.1) and 6% PEG6000 [w/v]} solutions were mixed (100 µL of each) and vigorously stirred for 20 min at 20°C. Then, 800 µL of 0.6 × precipitant solution was added to stop the crystal growth at a size of approximately 20 µm since, in our experience of SWSX, the size of *in meso* crystals is usually around 10–30 µm. The microcrystals were collected by centrifugation (2000 × *g*, 1 min, 20°C). After removal of the supernatant, 0.6 × precipitant solution (2.4 *M* Na-malonate [pH 3.1] and 3.6% PEG6000) was added and stored at 25°C. The microcrystal solution was adjusted to a final concentration of 3.2 *M* Na-malonate (pH 3.1) with an equal volume of 4 *M* of the same buffer. The density of the microcrystals was adjusted by adding 3.2 *M* Na-malonate (pH 3.1). The crystal suspension was scooped using a 400 or 600 µm loop (Protein Wave Corporation, Osaka, Japan), plunge-cooled into liquid nitro­gen, and stored in a UniPuck.

### 2.2. Data collection from lysozyme microcrystals with SWSX   

Data were automatically collected by the ZOO system on BL45XU at SPring-8 using the PILATUS3 6M detector (Broennimann *et al.*, 2006 ▸) with the sample held at 100 K. The ZOO system automatically measured the flux at each wavelength and calculated the X-ray dose prior to the diffraction measurement. It also automatically selected the exposure conditions, such as detector readout speed or X-ray transmission, according to the specified dose, and collected the data. The beam size for data measurement was 18 µm (H) × 20 µm (V) because it is better to use a beam size that matches the crystal size in order to obtain the highest possible signal-to-noise ratio. The entire cryo-loop was raster-scanned using X-rays in order to find the microcrystals. Normally, SWSX does not include three-dimensional centering; thus, the beam tends not to illuminate the crystal if a larger wedge size is chosen. Moreover, it is difficult to control the thickness of the media on the loop during the harvesting, especially when mounting *in meso* crystals using the glass sandwich method, and the misalignment during rotation becomes more pronounced. From our experience, greater rotation ranges than ±5° from the angle where the raster scan is conducted promotes this misalignment. Furthermore, when the crystals are densely mounted in the loop without gaps, a larger oscillation width forces diffraction from several crystals to overlap on the detector and the data processing works poorly. Assuming data collection in such ‘real’ cases, SWSX was performed for up to 200 crystals per loop with 10°/crystal (default in ZOO) based on the results of spot finding. The wavelength was set at 1.0, 1.4 and 1.7 Å, and data were collected with doses of 1, 2, 5, 10, 20 and 40 MGy per 10° wedge. More than 400 sub-datasets were collected under each condition (Tables 1 ▸ and 2 ▸). At BL45XU, the third-harmonic X-ray is detected at a wavelength of 1.89 Å. In low-dose experiments such as for 1 MGy, X-ray transmission needs to be extremely reduced with attenuators, and the negative effect of third-harmonic X-rays on the diffraction intensity increases. For this reason, we did not conduct experiments using longer wavelengths than 1.7 Å.

The dose per crystal of the data measured by the ZOO system was estimated by *RADDOSE-3D* v3.0.794 (Bury *et al.*, 2018 ▸). The calculation was performed with the following parameters: CELL 77.0 77.0 37.0 90.000 90.000 90.000, NRES 129, NMON 8, PATM S 10, SolventHeavyConc Na 2560, SolventFraction 0.36, Beam Type Gaussian, FWHM 20 18, and Collimation Rectangular 30 54. Hereafter, the ‘Max dose’ calculated by *RADDOSE-3D* is referred to as ‘dose’ (Tables 1 ▸ and 2 ▸). At BL45XU, the beam was defocused in the vertical direction to obtain this beam size. Therefore, the beam shape in the vertical direction was close to top-hat, and this parameter was used. In this study, the assessment and interpretation of the data is based on the assumption that the crystal is constantly irradiated with X-rays at the measured intensity and beam size during the data collection.

Indexing, integration and merging for each sub-dataset was performed by *XDS* (Version: 31 January 2020) using *KAMO*, which were then categorized into 18 groups with three incident wavelength (1.0, 1.4 and 1.7 Å) and six dose (1, 2, 5, 10, 20, and 40 MGy) combinations. For each group, unit-cell-based hierarchical clustering using *BLEND* (Foadi *et al.*, 2013 ▸) was applied, rejecting sub-datasets with non-equivalent unit-cell constants before merging, and resultant isomorphous sub-datasets were merged using *XSCALE*. All described steps were conducted automatically with *KAMO* (Yamashita *et al.*, 2018 ▸). Next, we created merged datasets using only the first half of the collected 10° data. Thus, for example, 5° data collected at a dose of 10 MGy could be generated from data collected at 20 MGy. These virtual 5° sub-datasets consisted of 18 groups, similar to the 10° sub-datasets, with different doses (0.5, 1, 2.5, 5, 10 and 20 MGy) (Tables 3 ▸ and 4 ▸). Degradation of intensity statistics was assumed to be caused by radiation damage as defined by absorbed dose, and any data rejections or corrections were not applied to the datasets.

### 2.3. S-SAD phasing   

#### 2.3.1. Phase determination by *SHELXC/D/E*   

For each merged dataset, S-SAD phasing was performed using *SHELX* (Sheldrick, 2010 ▸). *SHELXC* (Version 2016/1)/*SHELXD* (Version 2013/2) was used to determine the sulfur sites (*SHELXD* option: site = 10, cycle = 1000, *d*_min_ ≃ 1.7 Å for λ = 1.0 Å, 1.9 Å for λ = 1.4 Å and 2.2 Å for λ = 1.7 Å). *SHELXE* (Version 2019/1) was employed for phasing and density modification (*SHELXE* option: solvent contents 0.36, density modification 20 cycles, auto-build 3 cycles, *d*_min_ ≃ 1.19 Å for λ = 1.0 Å with 1 or 40 MGy dose, 1.17 Å for λ = 1.0 Å with 2 or 20 MGy dose, 1.15 Å for λ = 1.0 Å with 5 or 10 MGy dose, 1.41 Å for λ = 1.4 Å with all dose conditions, and 1.72 Å for λ = 1.7 Å with all dose conditions).

#### 2.3.2. Phasing with different number of sub-datasets   

To investigate the effect of the number of merged sub-datasets (referred to as ‘the number of sub-datasets’) on phasing, S-SAD phasing by *SHELX* was performed with several different numbers of merged datasets. The different numbers of sub-datasets were randomly extracted from all measurements at the same wavelength and dose, then scaled and merged by *XSCALE*. The number of sub-datasets was set to eight patterns (25, 50, 75, 100, 125, 150, 175 and 200 sets), and ten rounds of random dataset extraction and merging were independently conducted for each pattern. Since there were three different wavelengths and six different doses, in total 1440 merged datasets were generated for further investigation, which were used for the phase determination in *SHELX* as described above. Using each merged dataset and the program *SHELXC/SHELXD*, the sulfur sites in lysozyme were determined (*SHELXD* option: site = 8, cycle = 1000, *d*_min_ ≃ 1.7 Å for λ = 1.0 Å, 1.9 Å for λ = 1.4 Å, and 2.2 Å for λ = 1.7 Å). The determined heavy-atom positions were then used to obtain the phases after density modification using *SHELXE* (*SHELXE* option: solvent contents 0.40, density modification 20 cycles, auto-build 1 cycle, *d*_min_ = 1.15 Å for λ = 1.0 Å, 1.41 Å for λ = 1.4 Å, and 1.72 Å for λ = 1.7 Å). To evaluate the phases as close to the initial phases as possible, the number of cycles for density modification and model building were limited. In addition, the correct phase was prepared by the following procedure. Using the lysozyme model (PDBID: 1LYS), 30 cycles of jelly body refinement implemented in the program *CCP4/REFMAC5* (Murshudov *et al.*, 2011 ▸) were performed. Further refinement of *XYZ* coordinates and *B*-factors, and automatic water picking were conducted by *phenix.refine*. Considering the phases obtained from the refined model as the correct solution, the correlation coefficient (CC_map_) was estimated using *phenix.get_cc_mtz_pdb*.

### 2.4. SIRAS phasing of membrane protein YeeE with SWSX   

At SPring-8 beamline BL32XU, we achieved phase determination on *in meso* crystals of the membrane protein YeeE from a dataset obtained by SWSX using ZOO (Tanaka *et al.*, 2020 ▸). Absorbed dose and wedge size for data collection were set to 10 MGy and 7°, respectively, since a definitive guideline for phasing data collection was not clear at the time. The Bijvoet ratio of this sample in selenium-SAD at 0.9790 Å wavelength corresponded to 4.14%. In this structure analysis, selenium (Se) peak wavelength and native datasets from the YeeE *in meso* crystals were analyzed using SIRAS (single isomorphous replacement with anomalous scattering). Detailed information on crystallization and data collection are summarized by Tanaka *et al.* (2020 ▸) and Table 5 ▸ therein, respectively. Crystal sizes roughly corresponding to 30 µm and 10 µm (H) × 15 µm (V) beam, which was the maximum useable at beamline BL32XU, were used for raster scan and data collection. Here, we use the Se anomalous dispersion datasets obtained previously to examine how the number of sub-datasets affects the phase determination. Of the 376 Se anomalous dispersion sub-datasets collected, we performed hierarchical clustering based on the correlation coefficient of intensities (referred to as CC clustering) for 325 sub-datasets with equivalent unit-cell parameters. Next, *SHELX* was applied to the resulting 18 merged datasets, which were then used to determine the phase using SIRAS. The number of atoms in the heavy atom search was set to seven in *SHELXD* because YeeE contains seven Se atoms per protein monomer. We also performed SIRAS phasing using the native dataset (2.52 Å resolution). The heavy-atom sites were used as input data to *SHELXE* to obtain the initial phases (*SHELXE*: density modification 20 cycles, auto-tracing 5 cycles). The CC_map_ between the maps calculated with the phase from *SHELXE* and those from the model PDB was calculated using *phenix.get_cc_mtz_pdb*.

## 3. Results and discussion   

### 3.1. S-SAD phasing of lysozyme with SWSX   

More than 400 10° sub-datasets were collected from lysozyme crystals in combined patterns at incident wavelengths of 1.0, 1.4 and 1.7 Å, and doses of 1, 2, 5, 10, 20 and 40 MGy. Experimental phases were successfully determined for all dose conditions for data obtained at a wavelength of 1.7 Å, and the automated chain tracing function of *SHELXE* built more than 76% of the main chain model of the lysozyme. For the datasets obtained at 1.4 Å wavelength, the phases were successfully determined and the autotracing function built more than 76% of the main chain model, except for the data at a dose of 40 MGy. For the datasets collected at 1.0 Å, only those with a dose of 5 MGy resulted in successful phasing by S-SAD and more than 78% of the main chain was constructed; however, the correct phases were not obtained for the merged datasets with the other dose conditions. Statistics of the merged datasets collected at 1.0 Å are not shown.

**Figure 2:**
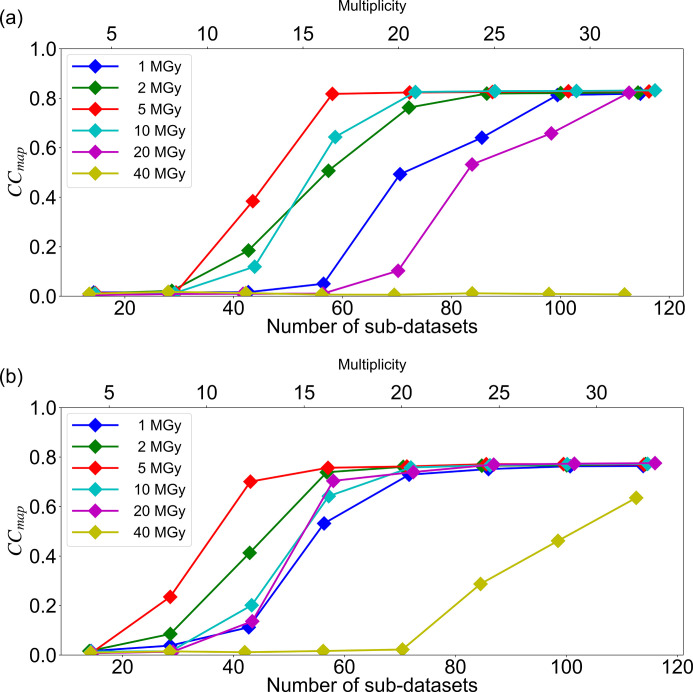
Correlation between the number of data merged for each dose and CC_map_ for (*a*) λ = 1.4 Å, (*b*) λ = 1.7 Å. Mean values of the correlation coefficient (CC_map_) derived from the phase determinations for ten randomly selected merged sub-datasets were plotted against the number of sub-datasets.

**Figure 3:**
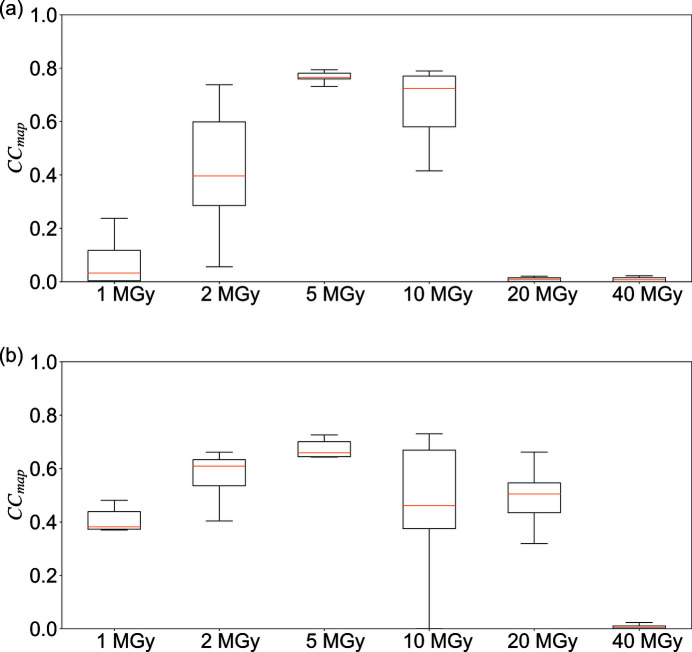
Correlation between each dose (1.0, 2.0, 5.0, 10.0, 20.0 and 40.0 MGy) of 100 merged 10° wedge data and the correlation coefficient (CC_map_) for (*a*) λ = 1.4 Å and (*b*) λ = 1.7 Å. Mean values are shown as orange lines.

To investigate the contribution of the number of sub-datasets in phase determination, CC_map_ for each dose was plotted against the number of sub-datasets (25, 50, 75, 100, 125, 150, 175 and 200) for two different wavelengths, 1.4 and 1.7 Å (Fig. 2 ▸). Each point on Fig. 2 ▸ shows the average CC_map_ value computed from the merged datasets created with data randomly extracted ten times with the same number of sub-datasets as the corresponding dataset, as described in the methods section. The plot clearly illustrates that a larger number of sub-datasets makes phase determination easier at any dose. The 10 MGy data at a wavelength of 1.4 Å and the 20 MGy data at a wavelength of 1.7 Å had a small number of original sub-datasets, so the behavior was not natural due to the lack of randomness of dataset extraction. CC_map_ for both wavelengths became higher as the number of sub-datasets increased, except for the 40 MGy dose at 1.4 Å. Moreover, at both wavelengths, sub-datasets collected at 5 MGy resulted in a better phase with the smallest number of sub-datasets, and then the next best doses identified were 2 and 10 MGy. At a wavelength of 1.4 Å, the anomalous multiplicity was about 30 for a series of 100 merged subsets, and at a wavelength of 1.7 Å, the anomalous multiplicity was about 20 for a series of 75 merged subsets. At all the doses, datasets collected at 1.7 Å yielded better phase with fewer sub-datasets compared with those collected at 1.4 Å simply due to the larger Bijvoet ratio of lysozyme in S-SAD at 1.7 Å wavelength, 1.76% (*f*′′ = 0.67), than that at 1.4 Å wavelength, 1.23% (*f*′′ = 0.47). To investigate the phasing efficiency, CC_map_ calculated with 100 sub-datasets was compared at different doses. At each dose condition, CC_map_ was calculated for ten randomly and independently extracted sub-datasets and depicted as box plots (Fig. 3 ▸). At any wavelength, mean and minimum CC_map_ values were highest for datasets resulting from 5 MGy, with the second-best dose being of 2 and 10 MGy, in agreement with the results shown in Fig. 2 ▸.

Figure 2 ▸ also demonstrates that the CC_map_ at 1 MGy was lower than that for other doses with the same number of merges, suggesting that the phasing becomes more difficult for reasons other than radiation damage. This effect could be due to the reduced total number of incident photons in the low-dose dataset compared with other datasets at the same merge number, and the diffraction signal also being reduced. As with the other doses, merging more subsets recovered CC_map_ in lower doses. It is noteworthy that the number of merges required for phase determination was only about twice that of 1 MGy compared with that of 5 MGy, even though the latter had five times as much signal as the 1 MGy dataset at 1.4 Å wavelength (Fig. 2 ▸). The 1.7 Å wavelength plot also shows similar behavior though the effect is smaller than at 1.4 Å. There are three possible reasons for this result. Firstly, low-dose data are more advantageous for phase determination because of the reduced radiation damage. Secondly, increasing the number of merges and multiplicity may augment the signal; therefore more multiplicity simply raises 〈*I*/σ*I*〉 and enhances the data accuracy required for phasing (Liu, Zhang *et al.*, 2011 ▸). Thirdly, an increment of multiplicity has the effect of reducing random errors and enhances the precision of the anomalous differences as previously reported (Liu, Chen *et al.*, 2011 ▸; Storm *et al.*, 2017 ▸). SWSX is more similar to the ‘multi-crystal’ method reported by Liu *et al.* (Liu, Zhang *et al.*, 2011 ▸; Liu *et al.*, 2013 ▸), a measurement strategy aimed at enhancing signals by merging multiple full-rotation datasets from multiple crystals. These three reasons roughly explain why 1 MGy data achieved the same quality of phase determination as 5 MGy data with at least twice the multiplicity. However, with the degree of improvement shown in the multi-dataset and ‘multi-crystal strategy’ reports, the jumps in phase improvements seen with twice the amount of data appear to be smaller than our results. Therefore, the effect of merging to reduce the random errors alone could not fully explain our results. We considered another factor, ‘dose averaging effect’, combined with the described positive effects of merging in SWSX, which will be discussed later.

**Figure 4:**
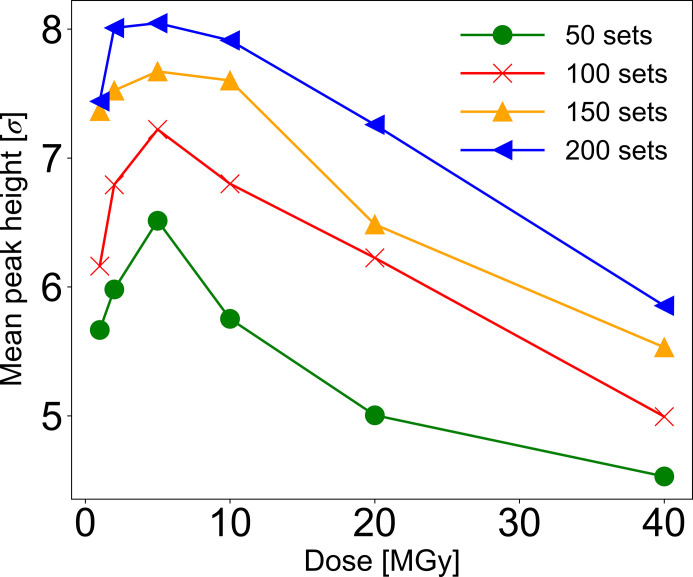
Correlation between each dose (1.0, 2.0, 5.0, 10.0, 20.0 and 40.0 MGy) of 50, 100, 150 and 200 10° sub-datasets and anomalous difference Fourier peak heights of sulfur sites of lysozyme for λ = 1.4 Å. The peak heights, analyzed by *ANODE*, above sigma level 4 were averaged and plotted against the dose for each number of sub-datasets.

The plots of 20 and 40 MGy high-dose data in Fig. 2 ▸ clearly show that phase determination becomes more difficult possibly due to severe radiation damage. There are two major steps in the phase determination by S-SAD. One is to determine the sulfur sites from the differences in the anomalous dispersion of the intensity data, and another is to calculate and refine the phases. Herein, the determination of the sulfur site prior to phase calculation was investigated. Sulfur sites determined for each merged dataset were evaluated using the software *ANODE* (Thorn & Sheldrick, 2011 ▸), which enables visualizing anomalous difference Fourier map peaks from phase information derived from existing protein coordinates. For merged datasets, averaged peak heights of anomalous difference Fourier maps from the sulfur atom were depicted as a series of plots for various doses and the number of sub-dataset conditions (Fig. 4 ▸). The result suggests that data collection with less than 10 MGy is preferable for determining heavy-atom sites. Nevertheless, increasing the number of merges improves the ease of determining the heavy-atom sites even at more than 10 MGy. Furthermore, at 1.7 Å, the behavior of the CC_map_ increment is also shown at 40 MGy dose. When all 298 sub-datasets were merged, phasing and main-chain tracing by *SHELXE* were successfully performed even with 40 MGy dose datasets (Table 2 ▸). As with the low-dose experiment, the reduction in random errors owing to multiple measurements did not completely explain the reason for the ability to determine the phase from the higher-dose data.

Another possible reason for the high probability of phase determination by merging at lower and higher dose is the averaging effect of the dose in SWSX. Despite Owen & Sherrell’s (2016 ▸) careful simulation of the relationship between structural changes and structure factors, we simply considered simulating the effect of radiation damage on macroscopic changes in structure factors in SWSX. An important consideration was the change in the temperature factor of the diffraction intensity. It is known to be a good approximation that, in addition to the intrinsic temperature factor of the crystal, there is an increase in the temperature factor of 1 Å^2^ per MGy due to global radiation damage during data collection (Kmetko *et al.*, 2006 ▸).

First, the intensity of the radiation-damaged reflections can be expressed by the following equation, with the temperature factor enlarged by radiation damage,
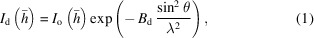
in which 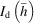 and 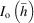 represent the reflection intensity with the radiation damage and the original reflection intensity including the intrinsic *B*-factor, respectively. As *B*_d_ increases, 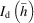 is affected in a θ-dependent manner. Finally, each independent reflection intensity is the mean value of multiple observations as
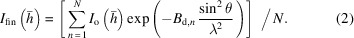
Taking the natural logarithm of both sides of equation (2),
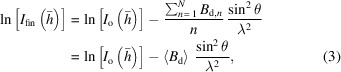
we obtain the well known form of the Wilson plot. As shown in equation (3), the acquired reflection intensity after damage can be expressed as a linear relationship with 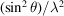 and the term 〈*B*_d_〉 correlates with the gradient of the Wilson plot. Equation (3) demonstrates that 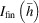 is defined by 〈*B*_d_〉 among equivalent measurements. As shown in Fig. 1 ▸(*c*), by collecting sub-datasets from multiple randomly oriented crystals, the starting angle of the data collection is random and the dose for observing a particular reflection intensity is also random for each crystal. The maximum dose is equal to the value set for collecting each sub-dataset. In the example in Fig. 1 ▸(*c*), 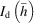 is included in the S1–S4 sub-datasets, which are collected with a dose of 9, 6, 2 and 1 MGy, respectively. If *B*_d_ is assumed to increase by 1 Å^2^ as absorbed dose increases by 1 MGy, the final 〈*B*_d_〉 is 4.5 Å^2^, a mean value of all *B*_d,*n*_ values.

**Figure 5:**
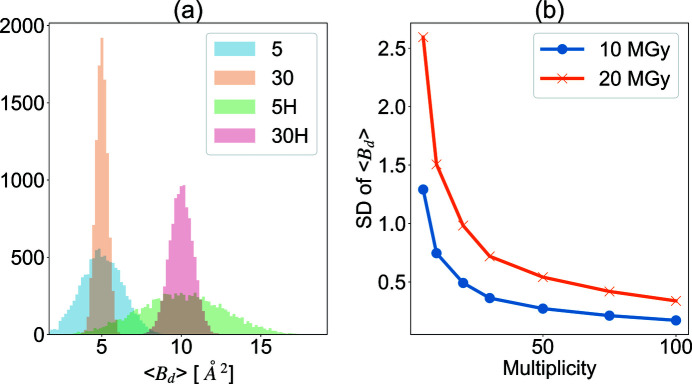
Simulation result of 〈*B*_d_〉 and its standard deviation (SD) in small-wedge synchrotron crystallography (SWSX) at doses of 10 and 20 MGy. (*a*) Histogram of 〈*B*_d_〉 of 10000 reflections where the multiplicity corresponded to 5 and 30. The ‘5’ and ‘30’, ‘5H’ and ‘30H’ in the legend indicate multiplicity of 5 and 30 for 10 MGy, and multiplicity of 5 and 30 for 20 MGy, respectively. (*b*) Multiplicity dependence of SD of the 〈*B*_d_〉 for 10 and 20 MGy.

Therefore, we simulated the relationship between multiplicity and distributions of 〈*B*_d_〉 and its standard deviation for 10000 independent reflections. Figure 5 ▸ shows the results of the simulation in the SWSX data collection with doses set to 10 MGy and 20 MGy. The histograms of 〈*B*_d_〉 demonstrate that the intensity after merging converges to half of the dose with higher multiplicity [Fig. 5 ▸(*a*)]. The standard deviation of *B*_d_, which is a component contributing to the variance of the reflection intensity from the true value, also decreases as the multiplicity increases [Fig. 5 ▸(*b*)]. Furthermore, the graphs also clearly show that the higher the dose during data collection, the wider the distribution of *B*_d_ for the same multiplicity. This also illustrates that it is easy to improve the precision of the data with a lower dose, even with a smaller number of crystals.

**Figure 6:**
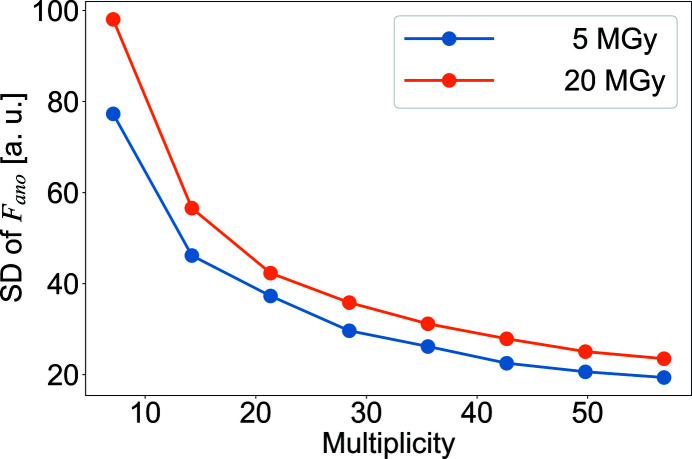
Standard deviation (SD) of observed anomalous difference in small-wedge synchrotron crystallography (SWSX). SD of the anomalous difference calculated from the observed structure factors using merged datasets collected at 1.4 Å wavelength; doses 5.0 MGy (blue) and 20.0 MGy (orange).

For comparison with this simulation, Fig. 6 ▸ plots the standard deviation of the anomalous differences of the structure factors for each number of merges at wavelength 1.4 Å. The behavior of the standard deviation of the anomalous difference in the observed structure factors against multiplicity appears to be similar to that of 〈*B*_d_〉 against multiplicity in the simulation in Fig. 5 ▸. This result shows that the effect of merging is rapidly obtained in a region where multiplicity is <200, and then the improvement in accuracy gradually increased. This feature is also consistent with the observed CC_map_ behaviors (Fig. 2 ▸). Our findings show that increasing the number of merges has the effect of reducing the ‘apparent dose’ in SWSX, and also improves the accuracy of the structure factor obtained. Hence, it is quite important to control the upper limit of the dose at the time of data collection for the faster convergence of 〈*B*_d_〉.

The effect of this dose averaging among merging subsets, both at low and high doses, improved the accuracy of the anomalous differences necessary for phase determination in our experiments. In other words, by using a large number of crystals in SWSX, and repeatedly collecting data at high doses, as opposed to multi-dataset or dose-slicing strategies, the combination of these positive effects of signal summation, reduction of random errors and dose averaging allows the structure to be determined even at extremely high doses. As a result of the overall effect of these factors, a dose of 5 MGy per crystal is the best quality data collection for SWSX with a 10° wedge.

The effect of changing wedge size with the same dose was investigated next for experimental phasing with SWSX by creating 5° subsets using the former half of 10° subsets. In this case, for example, the first 5° of 10° data collected with a total dose of 10 MGy will have a total dose of 5 MGy. Our goal was to understand how the phase-determinable dose systematically changes with an increasing number of incident photons per unit rotation angle. At a given total absorbed dose, a data set composed of 5° wedges is characterized by twice as many incident photons per oscillation width compared with a 10° wedge data set. Generally, a larger number of incident photons resulted in diffraction with higher resolution, while completeness and multiplicity of 10° wedge was twice as large as that of 5°. Therefore, in some cases, high intensity and low multiplicity measurements are preferred to acquire a higher-resolution dataset.

**Figure 7:**
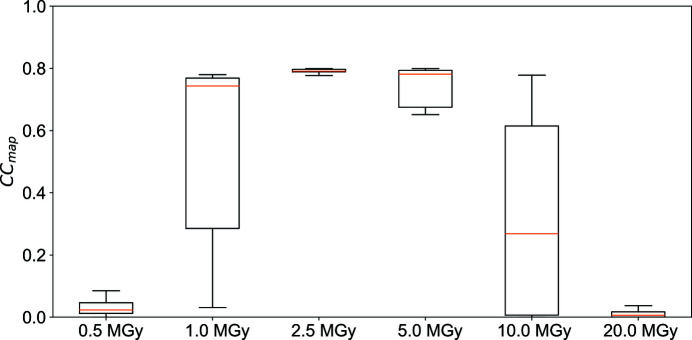
Correlation between each dose (0.5, 1.0, 2.5, 5.0, 10.0 and 20.0 MGy) of 200 merged 5° wedge data and the correlation coefficient (CC_map_) for λ = 1.4 Å. Mean values are shown as orange lines.

With the 10° sub-datasets, we were able to successfully determine the phase at 5 MGy by merging 100 sets, whereas with the 5° wedge data with the same number of crystals the phase determination was not successful. For 5° wedge data, merging 200 subsets with the same multiplicity of 10° wedge data finally enabled the phase determination; anomalous multiplicity roughly corresponded to 30. The mean CC_map_ for each dose (Fig. 7 ▸) revealed that data collection with 2–10 MGy resulted in successful phase determination at higher rate than for other dose conditions. This result is not directly comparable with Fig. 3 ▸ because the number of sub-datasets used was different from that of the 10° wedge data. Taken together, wedge size was not a significant factor, showing a very natural result concerning multiplicity by changing the wedge size. For the same number of sub-datasets, the multiplicity was halved compared with 10° in the 5° case; therefore, about twice the number of sub-datasets were needed to achieve phase determination. This result shows that increasing the intensity at the expense of multiplicity is not very effective for better phasing, and that increasing the multiplicity is more important. Moreover, these findings indicate that data should be collected with a larger wedge if the same number of crystals are used. However, as described in the previous sections, the probability that the beam and the crystal are not aligned will increase with the wedge size used in the measurements. Overall, it is necessary to perform the data collection with an optimal wedge size depending on the accuracy of the diffractometer and the beam size. In the automatic data collection by ZOO at SPring-8, the recommended upper limit of the wedge size for SWSX with 10–30 µm beam corresponds to 10°.

Since phasing was the goal, it was essential to increase the multiplicity more efficiently rather than increasing the number of photons per rotation angle to gain signal with the same dose, which agreed with the results reported by Liu, Chen *et al.* (2011 ▸).

### 3.2. Experimental phasing of membrane protein with SWSX   

**Figure 8:**
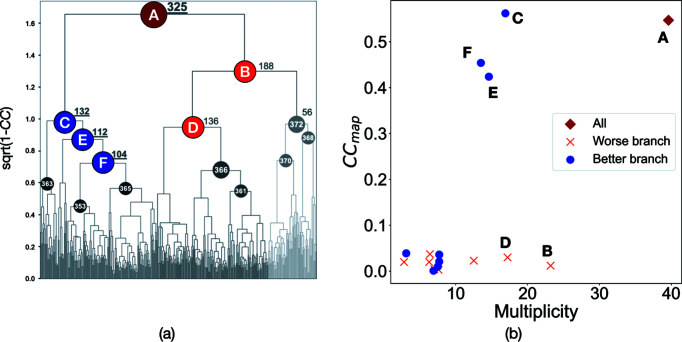
Summary of structure determination of the membrane protein YeeE. (*a*) Dendrogram of the hierarchical clustering based on the intensity CC with *KAMO*. Letters in circles correspond to cluster IDs of merged datasets. The number next to the cluster ID indicates the number of subsets at the corresponding node. (*b*) Relationship between the correlation coefficient (CC_map_) in phasing and data multiplicity. CC_map_ plotted against multiplicity in merged sub-datasets. Cluster IDs are also noted nearby each plot point using the same label as in (*a*).

In the structural study on the YeeE membrane protein, 17 merged datasets were obtained from the clustering using intensity-correlation [Fig. 8 ▸(*a*)]. *KAMO* automatically rejects outlier frames and data sets, and the results presented are after this polish. In an initial structure determination, only the top node whose cluster ID was A, which was obtained just by rejecting the outlier sub-datasets, was used for phasing and to obtain the final structure. However, other nodes with less merged sub-datasets were not investigated. Here, we demonstrate the phasing calculations with all merged datasets to investigate the power of the hierarchical clustering technique and the contribution of the number of sub-datasets in phasing. A CC_map_ was calculated from the map obtained from the experimental phases and refined model phases, and plotted against the number of sub-datasets [Fig. 8 ▸(*b*)]. There were two main branches from the top node, as shown in the dendrogram of CC clustering [Fig. 8 ▸(*a*)]. Phasing was successful for the merged datasets at a node with a cluster ID C, and failed for those in the other node of ID B. Under node C, the merged datasets comprising more subsets showed a higher CC_map_ value. In contrast, the CC_map_ values were not improved in any merged datasets under node B. These results illustrate two important features of SWSX. Firstly, clustering a large number of subsets by the CC of the intensity detects that there are two groups; one that is useful for phase determination and one that is not. For the data from one branch, phasing was successfully performed at a multiplicity of around 15, whereas for the data from the other branch phasing failed even though the multiplicity was greater than 20. Secondly, the plots for nodes C, E and F in Fig. 8 ▸(*b*) showed that the larger the number of sub-datasets in the better branch, the easier the phase determination becomes. This is supported by the S-SAD study of SWSX for lysozyme presented in the previous section.

Based on these results, for SWSX it is desirable to collect from larger wedges and from as many crystals as possible. In the example of YeeE structure determination, out of 325 wedges, more than half of the crystals were classified as branches that were not useful for phase determination. Since such ratios are not known prior to data measurement, collecting as many sub-datasets as possible will allow the best use to be made of the clustering technique for data selection. In addition, it is certain that more accurate phase information can be retrieved by increasing the number of useful sub-datasets, as observed in the results of lysozyme and the better branch of YeeE, which are of better quality. As well as hierarchical clustering, several methods of data selection have been proposed to increase the precision of the structure factors in the merging process (Zander *et al.*, 2016 ▸; Guo *et al.*, 2019 ▸).

## 4. Conclusion   

We have investigated the optimal dose when the wedge size is fixed at 10° in SWSX with respect to phase determination with S-SAD of lysozyme. As a result, it was shown that the most efficient phase determination can be achieved by data collection with an upper limit of ∼5 MGy. This value is actually close to the dose of diminishing returns for S-SAD phasing determined by Storm *et al.* using several hundred micrometre-sized crystals (Storm *et al.*, 2017 ▸).

The lysozyme S-SAD phasing study clearly showed that phasing becomes easier when the number of subsets is increased, regardless of the dose or wavelength. This may be due to a combination of the following factors: (1) repeated measurements enhance the signal-to-noise ratio of reflections, (2) increasing the multiplicity reduces random errors, and (3) merging a large number of sub-datasets may reduce the ‘apparent dose’ due to averaging (as shown in Section 3.1). Feature (3) is quite important for SWSX from the viewpoint of ‘dose control’. The simulation shown in Fig. 5 ▸ illustrates this clearly. We can see that the higher the multiplicity, the smaller the variance of *B*_d_, and the more precise the structure factor. In addition, it also clearly shows that the higher the dose in data collection, the larger the variance of *B*_d_ becomes for the same multiplicity. In other words, it is clear that data collection at higher doses works against obtaining accurate structure factors, especially when the number of crystals is limited.

We also found that clustering by the CC of intensity can be used to detect useful and non-useful groups in the phase determination of a real membrane protein sample. In addition, as with lysozyme, phasing became easier when the number of merges was large, as was the case for crystals useful for phasing. Therefore, it is important to collect as many subsets as possible, to select them and be able to merge many better datasets. However, as seen in Section 3.1, the possibility of low-dose SWSX should also be noted. From the results in Fig. 2 ▸, if we could gain twice as much multiplicity as if we had collected data at 5 MGy, we would have been able to make equivalent phase determinations at doses as low as 1 MGy. If, for some reason, the dose calculation is quite difficult, one strategy is to repeatedly collect low-dose sub-datasets. For example, this corresponds to data collection of five subsets of 1 MGy instead of collecting one subset of 5 MGy. This method is more time-consuming but safer from the viewpoint of radiation damage. Although common in the dose-slicing strategy, in the case of SWSX, collecting data multiple times from the same crystal increases the measurement time linearly with the number of data. In the case of dose-slicing, the amount of signal is simply reduced, so we need to pay close attention to ensuring the quality of data processing.

In this study, we analyzed SWSX with *in meso* crystals, so we used a 10° wedge case, since the crystals are densely mounted in a loop. As mentioned earlier, we chose this wedge size because an oscillation of more than 10° may cause the crystal to rotate out of the X-ray beam, or neighboring crystals may be irradiated, making data processing difficult. If the crystals were not closely mounted, the 20° wedge measurement would be expected to increase the multiplicity and facilitate the phase determination. For more than a 20° wedge data collection, it is important to know how to collect subsets with respect to the angle of the raster scan. Furthermore, raster scans must be done in several different orientations to align the X-rays with the crystal if data are collected from 10–30 µm crystals with a comparable-sized X-ray beam. The HITO module implemented in ZOO can automatically conduct these measurements by performing multiple raster scans at different orientations (Hirata *et al.*, 2019 ▸). It will take a longer time to perform multiple raster scans, and if there is enough machine time we can collect data with a larger wedge size. Of course, improved hardware performance, such as faster measurement of data subsets and shorter raster scan times, would expand the possibilities in SWSX.

For the first SWSX experiment, if 10–30 µm crystals are available, it will be effective to use a matching size X-ray beam and collect data from as many crystals as possible with 5 MGy dose over a range of ±5° from the raster scan angle. Making maximum use of collected sub-datasets, hierarchical clustering by intensity correlation to merge as many subsets as possible would enable and enhance phasing.

## Figures and Tables

**Table 1:** Crystallographic data statistics of 10° wedge with different doses, merge and S-SAD phasing statistics of lysozyme crystals for λ = 1.4 Å X-ray source: BL45XU; beam size: 18 µm (H) × 20 µm (V); detector distance: 140 mm; wedge/frame: 0.1°; and total wedge: 10°.

	1 MGy @ 1.4 Å	2 MGy @ 1.4 Å	5 MGy @ 1.4 Å	10 MGy @ 1.4 Å	20 MGy @ 1.4 Å	40 MGy @ 1.4 Å
Data collection						
Flux (photons s^−1^)	1.51×10^13^	1.51×10^13^	1.51×10^13^	1.51×10^13^	1.51×10^13^	1.51×10^13^
Average diffraction weighted dose/crystal (MGy)†	0.49	0.97	2.4	4.9	9.7	17.7
Max dose/crystal (MGy)†	1.1	2.1	5.3	10.6	21.3	38.8

Merging statistics						
Number of crystals	335	400	404	219	316	348
Space group	*P*4_3_2_1_2	*P*4_3_2_1_2	*P*4_3_2_1_2	*P*4_3_2_1_2	*P*4_3_2_1_2	*P*4_3_2_1_2
Unit-cell dimensions (Å)	*a* = *b* = 77.26, *c* = 38.42	*a* = *b* = 77.28, *c* = 38.43	*a* = *b* = 77.30, *c* = 38.44	*a* = *b* = 77.33, *c* = 38.45	*a* = *b* = 77.39, *c* = 38.51	*a* = *b* = 77.44, *c* = 38.50
Resolution (Å)	50–1.41 (1.50–1.41)	50–1.41 (1.50–1.41)	50–1.41 (1.50–1.41)	50–1.41 (1.50–1.41)	50–1.41 (1.50–1.41)	50–1.41 (1.50–1.41)
Completeness (%)	100.0 (100.0)	100.0 (100.0)	100.0 (100.0)	100.0 (100.0)	100.0 (100.0)	100.0 (100.0)
〈*I*/σ(*I*)〉	50.42 (4.04)	65.27 (5.55)	72.65 (7.47)	49.59 (4.95)	45.41 (3.31)	33.86 (2.12)
*R*_meas_ (%)	14.4 (273.5)	12.9 (246.7)	10.7 (171.2)	10.3 (143.3)	13.7 (212.9)	21.0 (297.2)
CC_1/2_	99.9 (95.4)	100.0 (97.4)	100.0 (98.4)	100.0 (96.6)	100.0 (93.7)	100.0 (85.9)
Anomalous correlation	15 (−2)	20 (−2)	26 (4)	20 (0)	16 (2)	11 (−2)
SigAno	1.073 (0.647)	1.211 (0.680)	1.273 (0.696)	1.039 (0.641)	0.954 (0.586)	0.855 (0.506)

S-SAD phasing	Success	Success	Success	Success	Success	Failure
Auto-build residues	127	121	108	99	102	–

† The values are as reported from *RADDOSE-3D* v3.0.794.

**Table 2:** Crystallographic data statistics of 10° wedge with different doses, merge and S-SAD phasing statistics of lysozyme crystals for λ = 1.7 Å X-ray source: BL45XU; beam size: 18 µm (H) × 20 µm (V); detector distance: 140 mm; wedge/frame: 0.1°; and total wedge: 10°.

	1 MGy @ 1.7 Å	2 MGy @ 1.7 Å	5 MGy @ 1.7 Å	10 MGy @ 1.7 Å	20 MGy @ 1.7 Å	40 MGy @ 1.7 Å
Data collection						
Flux (photons s^−1^)	8.17×10^12^	8.17×10^12^	8.17×10^12^	8.17×10^12^	8.12×10^12^	8.12×10^12^
Average diffraction weighted dose/crystal (MGy)†	0.49	0.98	2.5	4.8	9.8	20.5
Max dose/crystal (MGy)†	1.1	2.1	5.4	10.4	21.6	45.1

Merging statistics						
Number of crystals	353	453	358	395	218	298
Space group	*P*4_3_2_1_2	*P*4_3_2_1_2	*P*4_3_2_1_2	*P*4_3_2_1_2	*P*4_3_2_1_2	*P*4_3_2_1_2
Unit-cell dimensions (Å)	*a* = *b* = 77.28, *c* = 38.35	*a* = *b* = 77.28, *c* = 38.38	*a* = *b* = 77.29, *c* = 38.38	*a* = *b* = 77.33, *c* = 38.39	*a* = *b* = 77.38, *c* = 38.42	*a* = *b* = 77.46, *c* = 38.47
Resolution (Å)	50–1.72 (1.82–1.72)	50–1.72 (1.82–1.72)	50–1.72 (1.82–1.72)	50–1.72 (1.82–1.72)	50–1.72 (1.82–1.72)	50–1.72 (1.82–1.72)
Completeness (%)	100.0 (100.0)	100.0 (100.0)	100.0 (100.0)	100.0 (100.0)	100.0 (100.0)	100.0 (100.0)
〈*I*/σ(*I*)〉	72.42 (12.61)	92.87 (18.00)	97.73 (20.99)	91.26 (22.19)	62.44 (10.70)	50.27 (7.61)
*R*_meas_ (%)	11.1 (80.1)	10.0 (68.4)	7.9 (49.2)	9.3 (53.3)	8.7 (57.2)	13.2 (88.6)
CC_1/2_	100.0 (99.3)	100.0 (99.7)	100.0 (99.7)	100.0 (99.7)	100.0 (99.1)	100.0 (98.0)
Anomalous correlation	40 (10)	48 (16)	52 (20)	49 (19)	40 (2)	31 (9)
SigAno	1.533 (0.802)	1.812 (0.892)	1.873 (0.898)	1.842 (0.994)	1.245 (0.626)	2.629 (0.642)

S-SAD phasing	Success	Success	Success	Success	Success	Success
Auto-build residues	103	100	90	100	97	104

† The values are as reported from *RADDOSE-3D* v3.0.794.

**Table 3:** Crystallographic data statistics of 5° wedge with different doses, merge and S-SAD phasing statistics of lysozyme crystals for λ = 1.4 Å X-ray source: BL45XU; beam size: 18 µm (H) × 20 µm (V); detector distance: 140 mm; wedge/frame: 0.1°; and total wedge: 5°.

	1 MGy @ 1.4 Å of 5° wedge	2 MGy @ 1.4 Å of 5° wedge	5 MGy @ 1.4 Å of 5° wedge	10 MGy @ 1.4 Å of 5° wedge	20 MGy @ 1.4 Å of 5° wedge	40 MGy @ 1.4 Å of 5° wedge
Data collection						
Flux (photons s^−1^)	1.51×10^13^	1.51×10^13^	1.51×10^13^	1.51×10^13^	1.51×10^13^	1.51×10^13^
Average diffraction weighted dose/crystal (MGy)†	0.25	0.49	1.2	2.5	4.9	8.85
Max dose/crystal (MGy)†	0.6	1.1	2.7	5.3	10.7	19.4

Merging statistics						
Number of crystals	326	396	404	380	392	338
Space group	*P*4_3_2_1_2	*P*4_3_2_1_2	*P*4_3_2_1_2	*P*4_3_2_1_2	*P*4_3_2_1_2	*P*4_3_2_1_2
Unit-cell dimensions (Å)	*a* = *b* = 77.62, *c* = 38.42	*a* = *b* = 77.27, *c* = 38.42	*a* = *b* = 77.28, *c* = 38.44	*a* = *b* = 77.29, *c* = 38.44	*a* = *b* = 77.33, *c* = 38.48	*a* = *b* = 77.39, *c* = 38.48
Resolution (Å)	50–1.41 (1.50–1.41)	50–1.41 (1.50–1.41)	50–1.41 (1.50–1.41)	50–1.41 (1.50–1.41)	50–1.41 (1.50–1.41)	50–1.41 (1.50–1.41)
Completeness (%)	100.0 (100.0)	100.0 (100.0)	100.0 (100.0)	100.0 (100.0)	100.0 (100.0)	100.0 (100.0)
〈*I*/σ(*I*)〉	35.98 (3.03)	47.71 (4.53)	55.55 (6.66)	52.12 (7.26)	48.67 (5.90)	35.74 (3.67)
*R*_meas_ (%)	14.0 (244.9)	11.8 (206.0)	9.7 (144.1)	9.7 (117.4)	10.1 (116.3)	12.4 (138.7)
CC_1/2_	100.0 (91.9)	100.0 (95.3)	100.0 (97.7)	100.0 (97.9)	100.0 (97.3)	100.0 (94.4)
Anomalous correlation	12 (0)	15 (0)	20 (2)	20 (2)	20 (3)	14 (−2)
SigAno	0.959 (0.662)	1.053 (0.679)	1.128 (0.721)	1.108 (0.748)	1.027 (0.670)	0.870 (0.561)

† The values are as reported from *RADDOSE-3D* v3.0.794.

**Table 4:** Crystallographic data statistics of 5° wedge with different doses, merge and S-SAD phasing statistics of lysozyme crystals for λ = 1.7 Å X-ray source: BL45XU; beam size: 18 µm (H) × 20 µm (V) µm; detector distance: 140 mm; wedge/frame: 0.1°; and total wedge: 5°.

	1 MGy @ 1.7 Å of 5° wedge	2 MGy @ 1.7 Å of 5° wedge	5 MGy @ 1.7 Å of 5° wedge	10 MGy @ 1.7 Å of 5° wedge	20 MGy @ 1.7 Å of 5° wedge	40 MGy @ 1.7 Å of 5° wedge
Data collection						
Flux (photons s^−1^)	8.17×10^12^	8.17×10^12^	8.17×10^12^	8.17×10^12^	8.12×10^12^	8.12×10^12^
Average diffraction weighted dose/crystal (MGy)†	0.25	0.49	1.3	2.4	4.9	10.3
Max dose/crystal (MGy)†	0.6	1.1	2.7	5.2	10.8	22.6

Merging statistics						
Number of crystals	353	450	349	387	390	329
Space group	*P*4_3_2_1_2	*P*4_3_2_1_2	*P*4_3_2_1_2	*P*4_3_2_1_2	*P*4_3_2_1_2	*P*4_3_2_1_2
Unit-cell dimensions (Å)	*a* = *b* = 77.27, *c* = 38.35	*a* = *b* = 77.27, *c* = 38.38	*a* = *b* = 77.28, *c* = 38.37	*a* = *b* = 77.30, *c* = 38.37	*a* = *b* = 77.33, *c* = 38.40	*a* = *b* = 77.38, *c* = 38.44
Resolution (Å)	50–1.72 (1.82–1.72)	50–1.72 (1.82–1.72)	50–1.72 (1.82–1.72)	50–1.72 (1.82–1.72)	50–1.72 (1.82–1.72)	50–1.72 (1.82–1.72)
Completeness (%)	100.0 (100.0)	100.0 (100.0)	100.0 (100.0)	100.0 (99.9)	100.0 (100.0)	100.0 (100.0)
〈*I*/σ(*I*)〉	51.33 (9.34)	66.62 (13.59)	72.36 (16.67)	73.50 (21.40)	74.40 (17.56)	53.38 (12.45)
*R*_meas_ (%)	11.1 (79.4)	9.7 (65.1)	7.4 (44.2)	8.0 (40.6)	6.9 (33.1)	8.8 (41.0)
CC_1/2_	100.0 (98.8)	100.0 (99.4)	100.0 (99.6)	100.0 (99.7)	100.0 (99.7)	100.0 (99.3)
Anomalous correlation	27 (4)	38 (11)	43 (11)	42 (15)	53 (22)	39 (12)
SigAno	1.250 (0.790)	1.448 (0.836)	1.523 (0.835)	1.629 (1.034)	1.473 (0.746)	1.163 (0.673)

† The values are as reported from *RADDOSE-3D* v3.0.794.

**Table 5:** Crystallographic data statistics of YeeE for SIRAS analysis

X-ray source	SPring-8 BL32XU
Wavelength (Å)	0.9790
Wedge/frame (°)	0.1
Total wedge (°)	7
Number of sub-datasets	325
Resolution (Å)	47.85–2.51 (2.60–2.51)
Total reflections	938265 (80428)
Unique reflections	12576 (1217)
Multiplicity	74.6 (66.1)
Completeness (%)	99.97 (100.00)
Space group	*C*222_1_
*a,b,c* (Å)	73.60, 95.68, 101.45
*α, β, γ* (°)	90, 90, 90
〈*I*/_σ*I*_〉	13.8 (2.1)
*R*_meas_ (%)	0.516 (5.271)
