# Guidelines for *de novo* phasing using multiple small-wedge data collection. Corrigendum

**DOI:** 10.1107/S1600577522001655

**Published:** 2022-02-15

**Authors:** Seiki Baba, Hiroaki Matsuura, Takashi Kawamura, Naoki Sakai, Yuki Nakamura, Yoshiaki Kawano, Nobuhiro Mizuno, Takashi Kumasaka, Masaki Yamamoto, Kunio Hirata

**Affiliations:** a Protein Crystal Analysis Division, Japan Synchrotron Radiation Research Institute, 1-1-1 Kouto, Sayo, Hyogo 679-5198, Japan; b Life Science Research Infrastructure Group, RIKEN SPring-8 Center, 1-1-1 Kouto, Sayo-Cho, Sayo-gun, Hyogo 679-5148, Japan

**Keywords:** small-wedge synchrotron crystallography (SWSX), protein crystallography, radiation damage, *de novo* phasing, dose

## Abstract

A figure in the article by Baba *et al.* [(2021), *J. Synchrotron Rad.* **28**, 1284–1295] is corrected.

The *x*-axis ranges (0–120) shown in Fig. 2 on p. 1290 of the article by Baba *et al.* (2021 ▸) are incorrect. The correct figure with the *x*-axes in the range 0–200 is published here. ▸

## Figures and Tables

**Figure 2:**
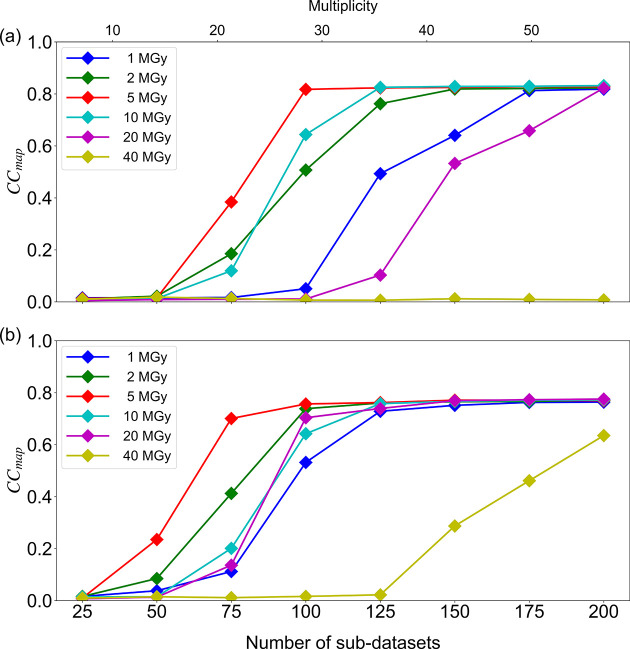
Correlation between the number of data merged for each dose and CC_map_ for (*a*) λ = 1.4 Å, (*b*) λ = 1.7 Å. Mean values of the correlation coefficient (CC_map_) derived from the phase determinations for ten randomly selected merged sub-datasets were plotted against the number of sub-datasets.
